# Supplementary material for: Sub-part-per-trillion test of the Standard Model with atomic hydrogen
Source: Nature. 2026 Feb 11;650(8103):845–51. doi: 10.1038/s41586-026-10124-3 (PMC12935534; doi:10.1038/s41586-026-10124-3)
Supplement: Supplementary file 1 — Supplementary Methods Sections 1 and 2 and Supplementary References. [file 41586_2026_10124_MOESM1_ESM.pdf]

nature portfolio

https://doi.org/10.1038/s41586-026-10124-3

Supplementary information

Sub-part-per-trillion test of the Standard Model with atomic hydrogen

In the format provided by the authors and unedited

Supplementary Methods for *Sub-part-per-trillion test of the Standard Model with atomic hydrogen*

Lothar Maisenbacher[1,4*], Vitaly Wirthl[1], Arthur Matveev[1], Alexey Grinin[1,5], Randolf Pohl[2], Theodor W. Hänsch[1,3], Thomas Udem[1,3]

[1]Max-Planck-Institut für Quantenoptik, Garching, Germany.
[2]Johannes Gutenberg-Universität Mainz, Mainz, Germany.
[3]Ludwig-Maximilians-Universität München, München, Germany.
[4]Present address: University of California, Berkeley, Berkeley, CA, USA.
[5]Present address: Northwestern University, Evanston, IL, USA.

*Corresponding author. E-mail: lothar.maisenbacher@mpq.mpg.de

1 Modeling

1.1 Modeling of atomic beam and fluorescence line shape

This section summarizes the modeling of the atomic beam and fluorescence line shape of the 2S-6P transition, which is described in detail in Sections 5.2 and 5.3 of [1], with some minor modifications introduced here.

First, the initial positions, directions, and speeds v of atomic trajectories originating from the nozzle are randomly sampled from, respectively, a uniform distribution over the nozzle orifice, a cosine law distribution, and the modified Maxwell-Boltzmann flux distribution for a given cut-off speed $v_{\text{cut-off}}$ (see main text). Trajectories not reaching the 2S-6P spectroscopy region are rejected, and the process is repeated until a set of N_{traj} is found ($N_{\text{traj}} = 1 \times 10^6$ and 4×10^6 for data groups G1A–G12 and G13–G14 (see Extended Data Table 3), respectively). These trajectories represent hydrogen atoms in the 1S ground level.

Second, for each of the 16 velocity groups ($i = 1, \ldots, 16$; see Extended Data Table 2) and each trajectory, the excitation probability $P_{2\text{S},i}$ to the metastable 2S level from the interaction with the preparation laser is found by numerically integrating the corresponding optical Bloch equations (OBEs) [2]. The ionization of the 2S level through single-photon absorption from the preparation laser limits the excitation probability. This process is also responsible for the lowered excitation probability for trajectories with low transverse velocity, as is visible in the transverse velocity distribution of the 2S atoms shown on the top of main text Fig. 3a. Importantly, the excitation probability is zero for trajectories that are too fast to both interact with the preparation laser and contribute to a given velocity group. Excluding these trajectories, $P_{2\text{S},i}$ ranges from $P_{2\text{S},1} = 2.1 \times 10^{-2}$ to $P_{2\text{S},16} = 6.4 \times 10^{-3}$ (see Extended Data Table 2).

Third, the excitation from the 2S level to the 6P level by the counter-propagating spectroscopy lasers beams and the subsequent decays are modeled with OBEs. Two different models, the quantum interference (QI) model (see Section 1.2) and light force shift (LFS) model (see Section 1.3), and corresponding sets of OBEs are used in this step. The OBEs of each model include signal equations that contain the expected fluorescence signal of the experimentally detected Lyman decays (split into their three spherical components for the QI model). The solution of the OBEs for each trajectory, velocity group and spectroscopy laser detuning Δ is found by interpolating precomputed numerical solutions of the OBEs on a three-dimensional, regular grid defined by trajectory speed (20 m/s... 1200 m/s), trajectory transverse angle (QI model; 0.5 mrad... 31.5 mrad) or transverse velocity (LFS model; 0 m/s... 38 m/s), and spectroscopy laser power (5 µW... 50 µW and 1 µW... 35 µW, respectively, for QI and LFS models and 2S-6P$_{1/2}$ transition; twofold lower power for 2S-6P$_{3/2}$ transition). We derive the OBEs using a computer algebra system [3] and numerically integrate them using an 8th-order explicit Runge-Kutta method [4] for the QI model and an implicit Runge-Kutta method (Radau IIA) of variable order [5] for the LFS model. The fluorescence signal for each velocity group, detuning, and decay (split into spherical components for the QI model) is found by summing the corresponding signal equation over all trajectories with the weight of each trajectory given by $P_{2\text{S},i}$. For the QI model, the three spherical components are weighted by their detection efficiency, given by simulated spatial detection efficiency of the two detectors, the component's radiation pattern, and the linear laser polarization angle θ_L, which defines the orientation of the radiation pattern relative to the detector cylinder. The LFS model, on the other hand, does not depend on spatial detection efficiency or θ_L, as it does not distinguish between spherical components. We use the signal from Lyman-ϵ decays only (summed over their weighted spherical components in case of the QI model) to mimic the experimental signal for each detector, velocity group, and detuning. Including the other Lyman decays, weighted by their detection efficiency, does not significantly influence the simulation corrections (except when static electric fields are included, see Section 1.4).

Fourth, the simulated fluorescence line shape for each detector and velocity group is analyzed in the same way as the experimental line scans (see Methods in the main text), i.e., by fitting either a Voigt or Voigt doublet line shape for data groups G1A–G12 or G13–G14, respectively. To ensure that the (relative) weight of the signal at each detuning j matches that of the experimental

data (where the weight $w_j = 1/y_j$ of signal y_j is the inverse square of the expected photon-number shot (Poissonian) noise $\sqrt{y_j}$ of the signal), a constant offset corresponding to the experimentally observed signal background is added to the simulated line shape. The QI shift ν_{QI} and the LFS ν_{LFS} are given by the resonance frequencies of the line shapes fitted to the QI and LFS simulations, respectively.

The line amplitudes A of the fitted line shapes are used to find the speed distribution of atoms contributing to the fluorescence signal for each velocity group (see Section 5.3.2 of [1]). To this end, the N_{traj} trajectories are sorted into 200 equal-width speed bins covering the range $v = 0\,\mathrm{m/s} \ldots 1000\,\mathrm{m/s}$, and, for each bin, the simulated line shape is calculated and fitted. This allows us to calculate the line amplitude of each velocity group for an arbitrary cut-off speed $v_{\text{cut-off}}$ by appropriately weighting the line amplitude of each bin. By comparing the simulated and experimental line amplitudes of the velocity groups for each line scan, we find the value of $v_{\text{cut-off}}$ that best describes the experimental data (see Extended Data Table 3). While not strictly necessary, we in practice first determine $v_{\text{cut-off}}$ (using a set of trajectories calculated using an initial value for $v_{\text{cut-off}}$) and then calculate a new set of N_{traj} trajectories using the optimal $v_{\text{cut-off}}$ for the rest of the analysis. The mean speed $\bar{v}$ and the root-mean-square speed $\bar{v}_{\mathrm{RMS}}$ of each velocity group are found from the weighted mean of the mean speed of the bins, using the line amplitudes as weights. We use the QI model with uniform spatial detection efficiency to determine $v_{\text{cut-off}}$, $\bar{v}$, and $\bar{v}_{\mathrm{RMS}}$; using the LFS model or the QI model with the detectors' spatial detection efficiencies instead does not give significantly different results.

For each experimental data group, the above steps are repeated using the appropriate optimal experimental parameters (see Extended Data Tables 3 and 5). This results in one value each of ν_{QI}, ν_{LFS}, $\bar{v}$, and $\bar{v}_{\mathrm{RMS}}$ for each velocity group and each detector (for ν_{QI} and ν_{LFS}) for every data group (see Extended Data Table 2 for the average value of $\bar{v}$ for each velocity group).

The uncertainty of the simulation corrections is found by varying the input parameters within their experimental limits. This is done for each input parameter individually while holding all other input parameters at their optimal values. The input parameters varied, their ranges of variation, and the resulting uncertainties are listed in Extended Data Table 5. When determining these uncertainties, we exclude any effects on ν_{e} from the variation in the mean speeds $\bar{v}$ caused by the variation of the input parameters. This allows us to distinguish the effect of a given input parameter variation on the light force, quantum interference, and second-order Doppler shifts from its common-mode effect on the values of $\bar{v}$, which, as explained above, are not sensitive to the specific underlying model. Instead, we include the uncertainty from the variation of $\bar{v}$ separately (see entry "Simulation of atom speeds" in main text Table 1 and Extended Data Table 4). We note that each input parameter variation contributes at most 12 Hz to this uncertainty.

1.2 Quantum interference (QI) model

The QI model underlies the QI simulations shown in this work and the estimations of the quantum interference shift given in main text Table 1 and Extended Data Table 4. It is described in detail in Section 2.3.2 of [1] (where it is referred to as "big model"). Briefly, the QI model contains all 148 levels coupled by the 2S→6P excitation and subsequent radiative, electric dipole-allowed (E1) decays. The level energies are taken from [6], which uses the equivalent of Eq. (1) in the main text to tabulate values. The dipole moments are calculated using non-relativistic wave functions as described in [7] (including reduced-mass corrections; relativistic corrections to the dipole moments are of order α^2 [8] and can be safely neglected here). All decay rates referenced in this work are derived from this model. Their values are (to 4 significant digits; dcy/s: decays per second): $\Gamma = 3.899\,\mathrm{MHz} = (2\pi \times 3.899)\,\mathrm{Mdcy/s}$, $\Gamma_{\text{e-1S}} = (2\pi \times 3.437)\,\mathrm{Mdcy/s}$, $\Gamma_{\mathrm{det}} = (2\pi \times 3.140)\,\mathrm{Mdcy/s}$ (corresponding to Lyman-ϵ decay), $\gamma_{\text{e-2S}} = (2\pi \times 462.0)\,\mathrm{kdcy/s}$, and $\gamma_{\mathrm{ei}} = (2\pi \times 153.6)\,\mathrm{kdcy/s}$ or $\gamma_{\mathrm{ei}} = (2\pi \times 306.4)\,\mathrm{kdcy/s}$ for the 2S-6P$_{1/2}$ or 2S-6P$_{3/2}$ transitions, respectively (with direct Balmer-δ decays contributing $(2\pi \times 151.6)\,\mathrm{kdcy/s}$ or $(2\pi \times 303.3)\,\mathrm{kdcy/s}$ to γ_{ei}). A signal equation for each (allowed) spherical component (σ^-, π, σ^+) of each decay between level manifolds with different principal quantum numbers is included, resulting in a total of 42 signal equations. This results in a total of 732 real-valued, nonzero coupled optical Bloch equations. The model includes the

two 6P fine-structure manifolds and terms describing cross-damping between their decays (cross-damping terms are included for all decays that share the same principal quantum numbers of both upper and lower levels). It therefore describes line shape distortions from quantum interference between excitation–decay paths that go through either 6P fine-structure manifold but start and end in the same level (the 2S-6P$_{1/2}$ and 2S-6P$_{3/2}$ transitions share the same initial level, but their excited levels belong to the $J = 1/2$ and $J = 3/2$ manifolds, respectively; see main text) [9, 10].

Because the different spherical components of the decays have different radiation patterns, a simulation of the spatial detection efficiency of the top and bottom detectors as a function of the photon emission direction is necessary to predict the fluorescence line shape observed in the experiment. To this end, a Monte Carlo particle tracing simulation of Lyman-ϵ photons and the photoelectrons ejected by them from the detector cylinder walls is employed, taking into account the different properties of the colloidal-graphite-coated walls and oxidized aluminum walls of the Faraday cage and the detector cylinder, respectively (see Section 4.6.6 of [1]). The uncertainty of the particle tracing simulation is estimated by varying the assumed transparencies of the meshes inside the detector cylinder, as meshes with different transparencies were used in the course of the experiment and as the mesh transparency is affected by the graphite coating, and by repeating the simulation (with $>1 \times 10^8$ photons used in each run) to account for the random nature of the simulation (see Extended Data Table 5). A similar particle tracing simulation was used to simulate the QI shifts in our previous measurement of the 2S-4P transition [11].

The top and bottom detectors are highly symmetric, except in one regard: the top detector has a solid aluminum cap on top of the detector cylinder, while the bottom detector has a graphite-coated mesh at the bottom of the detector cylinder to maintain the vacuum inside the cylinder (see main text Fig. 2b). The resulting difference in spatial detection efficiency leads to approximately 10 % larger QI shifts for the top detector than for the bottom detector.

In the perturbative limit [10], QI shifts are independent of laser power and vanish for a fluorescence detection covering the full (4π) solid angle. However, this is no longer the case when accounting for saturation and optical pumping, both included in our QI model, and we find two distinct effects to be of particular importance.

First, because of saturation, the ratio between the excitation rates of the off-resonantly coupled and the near-resonantly coupled 6P fine-structure components will increase with spectroscopy laser power. That is, the perturbing transition increases in strength relative to the perturbed transition, which in turn increases the QI shift.

Second, the back decay to the initial 2S level from the excited 6P levels introduces an asymmetry (with respect to the detuning from the probed transition) in the initial level's population, since the back decay is affected by QI distortions as well. This population asymmetry can then be imprinted on the population of the excited 6P levels by re-excitation and may be detected as signal upon decay. Importantly, the QI shifts from this optical pumping effect are independent of detection geometry. For our laser power range, they are found to be proportional to laser power with slopes of −20 Hz/µW and 21 Hz/µW for the 2S-6P$_{1/2}$ and 2S-6P$_{3/2}$ transitions, respectively.

While the second effect counteracts the saturation effect, overall the maximum QI shifts increase with laser power. For our laser power range and spatial detection efficiency, we find this increase to be approximately linear, amounting to 2.7 %/µW and 5.3 %/µW of the shifts in the perturbative limit for the 2S-6P$_{1/2}$ and 2S-6P$_{3/2}$ transitions, respectively. Likewise, the two effects lead to a dependence of the magic angle (the polarization angle θ_{L} for which the QI shifts are zero) on laser power, shifting from ≈54° in the zero-power limit to ≈52° at the highest laser powers used here.

Besides the QI effect, the presence of both 6P fine-structure manifolds in the model ensures that the dominant contribution to the ac-Stark shift of the 2S-6P transitions is included. It is estimated to be at most (i.e., at the highest spectroscopy laser powers used here) 41 Hz and −10 Hz for the 2S-6P$_{1/2}$ and 2S-6P$_{3/2}$ transitions, respectively. Contributions to the ac-Stark shift from off-resonant excitation of levels with $n \neq 6$, not included in the QI model, are estimated to contribute below 1 mHz in total for either transition [1].

1.3 Light force shift (LFS) model

The LFS model underlies the LFS simulations shown in this work and the LFS corrections given in main text Table 1 and Extended Data Table 4. It is introduced in the main text and described in detail in Section 3.4 of [1]. Briefly, it describes the state of the atoms in the combined basis of a simplified internal energy level scheme and the external momenta along the standing wave formed by the spectroscopy laser beams. The longitudinal motion is treated classically in the form of a time-dependent Rabi frequency in the rest frame of the atom, while the motion perpendicular to both the longitudinal direction and standing wave can be ignored here. The simplified level scheme consists of the initial $2S_{1/2}^{F=0}$, $m_F = 0$ level, either the excited $6P_{1/2}^{F=1}$, $m_F = 0$ or $6P_{3/2}^{F=1}$, $m_F = 0$ level, and a single 1S ground level, along with the (near-)resonant 2S→6P excitation and the 6P→2S and 6P→1S decays. Absorption or stimulated emission of a photon from or into either of the spectroscopy laser beams both changes the atom's internal level occupation and leads to a change in momentum along the beams by $\pm\hbar K_L$.

The effective rate of the 6P→2S back decay is set to γ_{ei}, which includes the direct and all indirect decays to the $2S_{1/2}^{F=0}$, $m_F = 0$ level. This is a good approximation because the indirect decays only contribute ≈1 % of γ_{ei} (see Tables 2.4 and 2.5 of [1]). All other decays of the 6P level are included in the effective 6P→1S decay rate (given by $\Gamma - \gamma_{ei}$), including those decays that would otherwise lead to 2S levels other than the $2S_{1/2}^{F=0}$, $m_F = 0$ level. This is a good approximation as those 2S levels are not resonantly coupled to the 6P levels and any population reaching them is effectively lost from the system.

The basis is split into two coupled subbases: one containing states before a momentum-changing 6P→2S back decay has taken place, and the other containing states after such a decay has changed the momentum by $\Delta p_{D,1}$ (picked from an appropriate distribution, see below). For the first subbasis, the rates of 6P→2S and 6P→1S decays are set to γ_{ei} and $\Gamma - \gamma_{ei}$, respectively, while for the second subbasis, they are set to zero and Γ. This effectively limits the number of back decays to one, as otherwise the required momentum basis grows with each subsequent back decay. Since $\gamma_{ei}/\Gamma \ll 1$, this is a good approximation, as confirmed by simulations including multiple subsequent back decays [1]. The subbases include coupled states with momenta ranging from $p_0 - 4\hbar K_L$ to $p_0 + 4\hbar K_L$ and $p_1 - 4\hbar K_L$ to $p_1 + 4\hbar K_L$, respectively, where p_0 is the initial momentum and $p_1 = p_0 + \Delta p_{D,1}$. The maximum momentum change ($\pm 4\hbar K_L$) that needs to be taken into account for the accuracy required here was found by varying the size of the basis [1]. This results in a total of 27 states (see Fig. 3.2 of [1] for a simplified visualization for $\pm 2\hbar K_L$) and 207 real-valued, nonzero coupled optical Bloch equations (OBEs), including 4 signal equations.

For each set of input parameters, the OBEs are numerically integrated for different values of $\Delta p_{D,1}$, and the resulting simulated fluorescence line shapes are weighted with the distribution of $\Delta p_{D,1}$ along the standing wave (Eq. (74) of [12]; note the different choice of quantization axis) and averaged. We use the Gaussian quadrature rule with 4 points to average over $\Delta p_{D,1}$. The averaged line shapes are fit in the same way as the experimental data to determine their resonance frequency, giving the LFS ν_{LFS} as shown in main text Fig. 3a, b. The width of the Bragg resonance of Fig. 3b is dominated by power broadening and time-of-flight broadening.

As a test, we use a perturbative analysis of the OBEs [13] at zero v_x and in the limit of small excited state population, which is approximately met here, to find $\nu_{LFS} \approx -|\Omega_0/(2\pi)|^2/(16\Delta\nu_{rec})$, where Ω_0 is the (angular) Rabi frequency of the 2S→6P excitation for each spectroscopy laser beam. Using an effective Rabi frequency of $\Omega_0 = (2\pi \times 109)$ krad/s to approximate the situation of an atom crossing the Gaussian spectroscopy laser beams, each with $P_{2S\text{-}6P} = 30\,\mu W$ ($15\,\mu W$) power for the 2S-$6P_{1/2}$ (2S-$6P_{3/2}$) transition, we find agreement within 60 Hz between this perturbative result and the full simulation.

Finally, we model the experimentally observed fluorescence line shape by summing up the line shapes of a set of atomic trajectories representing the atomic beam (see Section 1.1). This incoherent sum is valid because the incident transverse momentum states, as described by the Wigner function, are only mutually coherent over $2.6 \times 10^{-4}\hbar K_L$ in momentum space (this scale is not enhanced by propagation), which is much smaller than the momentum separation of $2\hbar K_L$ of the coupled states [1].

1.4 Modeling of dc-Stark shift

The mechanism underlying the dc-Stark shift is the mixing of the levels of interest with perturbing nearby levels of opposite parity by the electric field $\boldsymbol{E}$. The resulting new eigenstates are not only shifted in energy from their zero-field equivalents, but also no longer parity eigenstates. In particular, excitations from the initial 2S level of the 6S and 6D levels (which now have some admixture of the 6P level) become dipole allowed [1]. We note that the shift of the energy levels scales as n^7 [7].

Importantly, we do not directly determine the transition frequency between the levels of interest but instead measure and fit a fluorescence line shape to find the transition frequency. If excitations to perturbing levels contribute to this line shape, the dc-Stark shift $\Delta\nu_{\mathrm{dc}}$ of the transition frequency determined in this way is no longer adequately described by the energy shift of the levels of interest, but instead must be found from a fit to the line shape at field $\boldsymbol{E}$. Indeed, it can be shown within second-order perturbation theory (see Section 2.4 of [1]) that the dc-Stark shift of the center of mass of the mixed levels vanishes. However, this description is only adequate when the energy separations of the levels are much larger than their linewidths, which is not always the case here. In addition, a line shape fit is not necessarily equivalent to finding the center of mass of the mixed levels. Nevertheless, we may expect some cancellation and indeed observe this for the 2S-6P$_{3/2}$ transition (see below).

To investigate this, we simulate corresponding line shapes by extending our QI model (see Section 1.2) to include mixing terms from a static electric field with strength $E = |\boldsymbol{E}|$, either parallel or perpendicular to the quantization axis (set by the linear spectroscopy laser polarization), the mixed 6S and 6D levels, and their decays along with the necessary additional intermediate levels. The simulation also includes the field-induced mixing of the 2S levels with the 2P levels, but the resulting shift of the initial 2S level (which we find to be $0.4\,\mathrm{Hz/(V/m)^2}$) is negligible here. Because we treat the electric field as a perturbation, the relevant 6P levels (for which $F = 1$) only mix with levels with $F \neq 1$ for the case of electric field parallel to the quantization axis, and only with levels with $F \neq 0$ for the perpendicular case. This results in a total of 2070 and 5880 real-valued, nonzero coupled optical Bloch equations (OBEs) for the parallel and perpendicular cases, respectively. Due to the resulting computational cost, we only calculate single atomic trajectories, adding the experimentally observed Doppler broadening by convolving the simulated line shape with a Gaussian, except for the case of the 2S-6P$_{3/2}$ transition and electric field perpendicular to the quantization axis (see below), where we average over a set of trajectories representing the atomic beam. As for the QI simulation, the three spherical components of each decay are weighted by their detection efficiency and summed up. Since the laser polarization lies in the y-z-plane in the experiment (see main text Fig. 2b), an electric field along the x-direction is purely perpendicular to the quantization axis (i.e., $\beta_{\mathrm{dc},x} \equiv \beta_{\mathrm{dc},\perp}$), while electric fields along the y- and z-directions can be decomposed into components parallel and perpendicular to the quantization axis (i.e., $\beta_{\mathrm{dc},y} = \cos^2(\theta_{\mathrm{L}})\beta_{\mathrm{dc},\perp} + \sin^2(\theta_{\mathrm{L}})\beta_{\mathrm{dc},\parallel}$ and $\beta_{\mathrm{dc},z} = \sin^2(\theta_{\mathrm{L}})\beta_{\mathrm{dc},\perp} + \cos^2(\theta_{\mathrm{L}})\beta_{\mathrm{dc},\parallel}$). Note that while this decomposition is an approximation because the line shapes are different for parallel and perpendicular fields, we find reasonable or better agreement with the experimental data (see below). The simulations are performed for both the stray-field regime ($E < 1\,\mathrm{V/m}$) and the bias-field regime ($E = 10\,\mathrm{V/m} \ldots 45\,\mathrm{V/m}$) as defined in the Methods in the main text.

For the 2S-6P$_{1/2}$ transition, the perturbation to its excited $6\mathrm{P}_{1/2}^{F=1}$, $m_F = 0$ level is from mixing with the $6\mathrm{S}_{1/2}^{F=0}$ and $6\mathrm{S}_{1/2}^{F=1}$ levels, which are higher in energy by 34 MHz and 41 MHz, respectively, and, to a lesser degree, the $6\mathrm{D}_{3/2}^{F=1}$ and $6\mathrm{D}_{3/2}^{F=2}$ levels, which are higher in energy by 405 MHz, approximately the 6P fine-structure splitting [6]. All perturbing levels are therefore well outside the natural and experimental linewidth and we expect the dc-Stark shift of the 2S-6P$_{1/2}$ transition to be given by the shift of the $6\mathrm{P}_{1/2}^{F=1}$, $m_F = 0$ level (and the much smaller shift of the initial 2S level). Likewise, while the line of the transition will shift, its line shape will remain well-described by a Voigt line shape, as observed in the experiment (see Extended Data Fig. 3a). From second-order perturbation theory for the shift of the $6\mathrm{P}_{1/2}^{F=1}$, $m_F = 0$ level, we obtain $\beta_{\mathrm{dc},\parallel} = -1.75\,\mathrm{kHz/(V/m)^2}$ and $\beta_{\mathrm{dc},\perp} = -1.51\,\mathrm{kHz/(V/m)^2}$ for the static electric field parallel and perpendicular to the

quantization axis, respectively. The line shape simulations, which reproduce the experimental line shape well (see solid lines in Extended Data Fig. 3a), give values for $\beta_{\mathrm{dc},\parallel}$ and $\beta_{\mathrm{dc},\perp}$ that agree within 4 % (where spectroscopy laser powers and atom speed and transverse velocity were varied) with the perturbative values for both the stray- and bias-field regimes. Experimentally (for $\theta_{\mathrm{L}} = 56.5°$), we find $\beta_{\mathrm{dc},x} = -1.469(4)\,\mathrm{kHz/(V/m)^2}$, $\beta_{\mathrm{dc},y} = -1.530(3)\,\mathrm{kHz/(V/m)^2}$, and $\beta_{\mathrm{dc},z} = -1.707(7)\,\mathrm{kHz/(V/m)^2}$ (statistical uncertainties only), which agree within 4 % with the bias-field regime simulations (see Extended Data Fig. 3c for a measurement of $\beta_{\mathrm{dc},x}$ along with the simulation). We choose to use the experimental values of β_{dc} to determine the dc-Stark shift from stray electric fields for the 2S-6P$_{1/2}$ transition, expanding their uncertainty to cover both stray- and bias-field regimes and theory and simulation results ($\beta_{\mathrm{dc},x} = -1.47(4)\,\mathrm{kHz/(V/m)^2}$, $\beta_{\mathrm{dc},y} = -1.53(5)\,\mathrm{kHz/(V/m)^2}$, and $\beta_{\mathrm{dc},z} = -1.71(5)\,\mathrm{kHz/(V/m)^2}$).

For the 2S-6P$_{3/2}$ transition the situation is quite different. This is because the perturbation of its excited $6\mathrm{P}_{3/2}^{F=1}$, $m_F = 0$ level is dominated by the $6\mathrm{D}_{3/2}^{F=2}$ and $6\mathrm{D}_{3/2}^{F=1}$ levels, which are only separated in energy by 57 kHz and −469 kHz, respectively [6]. They are therefore well within the natural linewidth of the 2S-6P$_{3/2}$ transition, and the observed dc-Stark shift of the transition frequency determined from the line shape is not expected to correspond to the shift of the $6\mathrm{P}_{3/2}^{F=1}$, $m_F = 0$ level. Because of the strong mixing resulting from the small energy separation, we expect the line shape to be strongly perturbed, and indeed we experimentally observe a splitting of the line shape into two distinct components (see Extended Data Fig. 3b). We use a Voigt doublet line shape to fit the experimental and simulated line shape. The other perturbing levels, $6\mathrm{S}_{1/2}^{F=0}$ and $6\mathrm{S}_{1/2}^{F=1}$ (lower in energy by 365 MHz and 371 MHz, respectively), and $6\mathrm{D}_{5/2}^{F=2}$ (higher in energy by 135 MHz), are again well-separated compared to the linewidth and mix much less strongly with the $6\mathrm{P}_{3/2}^{F=1}$, $m_F = 0$ level.

We first consider the case of the electric field perpendicular to the quantization axis, for which both the $6\mathrm{D}_{3/2}^{F=2}$ and the $6\mathrm{D}_{3/2}^{F=1}$ levels are mixed within the linewidth. Our simulation (solid lines in Extended Data Fig. 3b) reproduces the splitting of the experimental line shape well and describes the resulting components' relative amplitudes reasonably well within ≈20 %. The simulation of a single trajectory without Doppler broadening and at a high bias field (green dashed line in Extended Data Fig. 3b) reveals a more complex substructure. In the bias-field regime, the simulations nevertheless result in a quadratic dc-Stark shift with $\beta_{\mathrm{dc},\perp} = -0.55(3)\,\mathrm{kHz/(V/m)^2}$ (orange diamonds and dashed line in Extended Data Fig. 3d). This agrees within 5 % with the experimental value of $\beta_{\mathrm{dc},x} = -0.573(7)\,\mathrm{kHz/(V/m)^2}$ (statistical uncertainty only; see blue circles and solid line in Extended Data Fig. 3d for a single measurement). Both values agree within 15 % with our second-order perturbation theory result of $\beta_{\mathrm{dc},\perp} = -0.50\,\mathrm{kHz/(V/m)^2}$ for the shift of the $6\mathrm{P}_{3/2}^{F=1}$, $m_F = 0$ level when the mixing with the $6\mathrm{D}_{3/2}^{F=2}$ and $6\mathrm{D}_{3/2}^{F=1}$ levels is ignored, i.e., assuming that there is no net contribution from levels within the linewidth. This can be understood as the line shape fit approximately finding the center of mass of the (split) line shape and thereby cancelling the shift from the perturbing levels causing the splitting. We use $\beta_{\mathrm{dc},\perp} = -0.55(3)\,\mathrm{kHz/(V/m)^2}$ as our estimate in the bias-field regime.

In the stray-field regime, where the splitting is not resolved, the simulations reveal a more complex behavior. There is a substantial dependence of $\beta_{\mathrm{dc},\perp}$ on the experimental parameters of each trajectory, especially on the transverse velocity, which we attribute to the interplay of line splitting due to the electric field and due to the Doppler shift. This is addressed by averaging over multiple trajectories representing the atomic beam, as explained above. Most strikingly, $\beta_{\mathrm{dc},\perp}$ strongly depends on which Lyman decay is observed (with the dc-Stark shift's quadratic behavior maintained for each decay within the stray-field regime), e.g., we find $1.39\,\mathrm{kHz/(V/m)^2}$ for Lyman-ϵ and $-17.7\,\mathrm{kHz/(V/m)^2}$ for Lyman-α decays (at $P_{\text{2S-6P}} = 15\,\mu\mathrm{W}$), which approximately constitute 97 % and 1.5 % of the fluorescence signal, respectively. We address this by summing up the Lyman decays with their estimated detection efficiency (see Fig. B1 of [1]). Overall, we estimate $\beta_{\mathrm{dc},\perp}$ in the stray-field regime as $0.56(75)\,\mathrm{kHz/(V/m)^2}$ (note the opposite sign to $\beta_{\mathrm{dc},\perp}$ in the bias-field regime).

Next, we consider the simpler case of the electric field parallel to the quantization axis, for which only the $6\mathrm{D}_{3/2}^{F=2}$ level is mixed within the linewidth. The agreement between the simulated and experimental line shapes is similar to the perpendicular case. However, as opposed to the perpendicular case, the simulated values of $\beta_{\mathrm{dc},\perp}$ show no strong dependence on the experimental parameters or on which Lyman decay is detected. Importantly, there is no drastically different behavior for the stray- and bias-field regimes, which we attribute to the presence of only one mixed level within the linewidth. Overall, we find $\beta_{\mathrm{dc},\parallel} = -0.48(5)\,\mathrm{kHz}/(\mathrm{V/m})^2$. Remarkably, this simulated value is much smaller than the second-order perturbation theory result of $\beta_{\mathrm{dc},\parallel} = -33.6\,\mathrm{kHz}/(\mathrm{V/m})^2$ for the shift of the $6\mathrm{P}_{3/2}^{F=1}$, $m_F=0$ level when all perturbing levels are included, but in excellent agreement with the result ($\beta_{\mathrm{dc},\parallel} = -0.48\,\mathrm{kHz}/(\mathrm{V/m})^2$) when the mixing with the $6\mathrm{D}_{3/2}^{F=2}$ level is ignored, again demonstrating the strong cancellation of the shift when finding the center of mass of the split line.

Using the bias-field regime value of $\beta_{\mathrm{dc},\perp}$ from above, we find the simulated values of $\beta_{\mathrm{dc},y}$ and $\beta_{\mathrm{dc},z}$ in the bias-field regime (for $\theta_{\mathrm{L}} = 56.5°$) to be $-0.53(3)\,\mathrm{kHz}/(\mathrm{V/m})^2$ and $-0.50(4)\,\mathrm{kHz}/(\mathrm{V/m})^2$, respectively, showing good agreement with the experimental value of $\beta_{\mathrm{dc},y}$ ($-0.540(5)\,\mathrm{kHz}/(\mathrm{V/m})^2$) and reasonable agreement for $\beta_{\mathrm{dc},z}$ ($-0.422(18)\,\mathrm{kHz}/(\mathrm{V/m})^2$). Likewise, we find the simulated values of $\beta_{\mathrm{dc},y}$ and $\beta_{\mathrm{dc},z}$ in the stray-field regime to be $0.24(52)\,\mathrm{kHz}/(\mathrm{V/m})^2$ and $-0.16(24)\,\mathrm{kHz}/(\mathrm{V/m})^2$, respectively. These two values, along with the stray-field-regime value of $\beta_{\mathrm{dc},x} \equiv \beta_{\mathrm{dc},\perp}$, are used in the determination of the dc-Stark shift of the 2S-$6\mathrm{P}_{3/2}$ transition. The Pearson correlation coefficients between ($\beta_{\mathrm{dc},x}, \beta_{\mathrm{dc},y}$), ($\beta_{\mathrm{dc},x}, \beta_{\mathrm{dc},z}$), and ($\beta_{\mathrm{dc},y}, \beta_{\mathrm{dc},z}$) are $r = 0.99$, $r = 0.97$, and $r = 0.95$, respectively.

2 Corrections and uncertainties

2.1 Second-order Doppler shift

The second-order Doppler shift (SOD) leads to an apparent shift of the atom's transition frequency ν in the laboratory frame of reference (see Section 2.2.3 of [1]). The average value of the shift for each velocity group with root-mean-square speed $\bar{v}_{\mathrm{RMS}}$ is given by

$$\Delta\nu_{\mathrm{SOD}} = -\frac{\nu}{2}\left(\frac{\bar{v}_{\mathrm{RMS}}}{c}\right)^2 . \tag{1}$$

The transition frequency ν is identical at the required level of accuracy for the two 2S-6P transitions probed here and is known with much lower uncertainty than needed here. $\bar{v}_{\mathrm{RMS}}$ and $\Delta\nu_{\mathrm{SOD}}$ range over 270 m/s. . . 67 m/s and −297 Hz. . . −18 Hz, respectively, for the different velocity groups.

Since the magnitude of the (in this geometry always negative) SOD increases (quadratically) with speed, it leads to an apparent negative Doppler slope of $\kappa = -1.7\,\mathrm{Hz}/(\mathrm{m/s})$ in the (linear) Doppler shift extrapolation if not accounted for. Here, we first correct the resonance frequency ν_0 of each velocity group of each line scan by subtracting $\Delta\nu_{\mathrm{SOD}}$, with $\bar{v}_{\mathrm{RMS}}$ determined with our atomic beam simulation, and subsequently performing the Doppler shift extrapolation. The effect of the removed apparent Doppler slope outweighs the shift of the individual velocity groups, and the correction applied to the Doppler-free transition frequencies $\nu_{1/2}$ and $\nu_{3/2}$ amounts to −0.15(1) kHz and −0.14(1) kHz, respectively (see Extended Data Table 5 for the uncertainty estimation). The correction is highly correlated ($r = 0.98$) between the 2S-$6\mathrm{P}_{1/2}$ and 2S-$6\mathrm{P}_{3/2}$ transitions.

2.2 Blackbody-radiation (BBR)-induced shift

We calculate the BBR-induced ac-Stark shifts of the 2S and 6P levels following [14] but include fine and hyperfine structure and the Lamb shift for levels with $n \leq 10$. However, because the resulting frequency splittings of the 2S and 6P levels (at most 10 GHz) are far below the peak of the BBR spectrum near room temperature (∼20 THz), this inclusion has a negligible effect at

our level of accuracy, and we find a difference of below 1 Hz to the shifts at a BBR temperature of 300 K given in Table 1 of [14]. At 290(10) K, which we estimate as the temperature of the BBR in the 2S-6P spectroscopy region, we calculate the BBR-induced shift of the 2S-$6P_{1/2}$ and 2S-$6P_{3/2}$ transitions to be −0.28(1) kHz. $\nu_{1/2}$ and $\nu_{3/2}$ have been corrected for the shift by subtracting this value, with the correction assumed to be fully correlated between the transitions. The BBR-induced broadening of the transitions, found to be 1.3 kHz at 300 K in [14], is negligible here.

2.3 Zeeman shift

The Zeeman shift of the observed transition frequency for a magnetic flux density B_x along the spectroscopy laser beams is given by (see Section 6.2.4.3 of [1])

$$\Delta\nu_{\text{Zeeman}} = \frac{S_3}{S_0}\frac{g_F \mu_{\text{B}} B_x}{h}. \tag{2}$$

$g_F = 1/3$ ($g_F = 5/3$) is the g-factor of the $6P_{1/2}^{F=1}$ ($6P_{3/2}^{F=1}$) level, and μ_{B} is the Bohr magneton (note that $g_F = 0$ for the $2S_{1/2}^{F=0}$ level). S_3/S_0 is the residual circularly polarized light fraction of the spectroscopy laser beams, where S_0 is the total intensity and S_3 the intensity difference between right and left circularly polarized light [15, 16].

The magnetic field in the 2S-6P spectroscopy region is minimized with three orthogonal pairs of Helmholtz coils outside the vacuum chamber, and a single-layer, high-permeability metal (mumetal) shield inside the vacuum chamber. All components inside this shield are made from nonmagnetic materials. B_x was measured to be below 1 mG within a 5 mm-radius sphere centered in the spectroscopy region. The absolute value of S_3/S_0 in the spectroscopy region was monitored in situ by measuring the polarization of the light backcoupled into the fiber of the active fiber-based retroreflector, as detailed in [15, 16]. For the data set presented here, $|S_3/S_0|$ is 4.2(1.3) % on average (standard deviation over the data groups in parentheses). No polarization data are available for a small number of line scans (approximately 9 %), and we instead use the upper limit of $|S_3/S_0|$ of 10 % from the available data.

Using the value $|S_3/S_0|$ of each line scan (or the upper limit where necessary), and the maximum value of B_x as given above, results in upper limits for the Zeeman shifts of $|\Delta\nu_{\text{Zeeman}}| =$ 0.02 kHz and $|\Delta\nu_{\text{Zeeman}}| =$ 0.11 kHz for the 2S-$6P_{1/2}$ and 2S-$6P_{3/2}$ transitions, respectively. We include these upper limits as uncertainties for $\nu_{1/2}$ and $\nu_{3/2}$. The uncertainties are assumed to be fully correlated between the 2S-$6P_{1/2}$ and 2S-$6P_{3/2}$ transitions.

2.4 Pressure shift

The collision of hydrogen atoms with nearby particles during the 2S→6P excitation leads to a pressure shift of the observed transition frequency. We distinguish between, on the one hand, intra-beam collisions with other hydrogen atoms (either in the 1S or 2S level) or hydrogen molecules in the atomic beam, and, on the other hand, collisions with particles from the background gas. The particles in the atomic beam are assumed to be at the temperature of the nozzle T_{N}, and the background gas is taken to be at room temperature.

We estimate the number of 1S atoms and hydrogen molecules leaving the nozzle per second in the direction of the 2S-6P spectroscopy region to be, respectively, 1.6×10^{16} atoms/s and 1.8×10^{16} molecules/s, with the latter corresponding to a flux density of 1.4×10^{17} molecules/(s/m^2) at the spectroscopy laser beams (see Section 4.5.2.3 of [1]). The background gas pressure within the spectroscopy region is estimated (from pressure measurements and conductance simulations) to be 2×10^{-7} mbar and is dominated by molecular hydrogen.

A Monte Carlo simulation of the pressure shift expected for the 2S-$6P_{1/2}$ and 2S-$6P_{3/2}$ transitions from intra-beam collisions with other hydrogen atoms, using the approximate parameters and geometry of the 2S-6P measurement, has been done in [17], based on recent calculations of the relevant van der Waals interaction coefficients C_6 [18, 19]. Scaling these results to the tenfold

lower flux of hydrogen atoms actually used here, we find the magnitude of this contribution to the pressure shift to be below 1 Hz for both transitions and for all velocity groups.

To estimate the pressure shift from molecular hydrogen, we evaluated the collisional shift cross section and then computed the shift using the estimated pressure and velocity distribution of molecules. The velocity-dependent collisional shift cross section was calculated using the S-matrix approach [20], with the matrix elements found by numerical solution of the Schrödinger equation describing evolution of atomic wave function during the collision and subsequently averaged in the collision parameter space by Monte Carlo integration. Finally, the pressure shift for the experimental conditions is calculated from the cross sections with a Monte Carlo simulation. We use a simplified model of the hydrogen molecule which takes into account both the van der Waals interaction with the perturbed hydrogen atom and the quadrupole electric field of the molecule [21]. The evaluation of the van der Waals interaction is complicated by the presence of dipole-allowed transitions in the hydrogen molecule with energy close to the 6P-1S transition in atomic hydrogen. To calculate the non-resonant van der Waals interaction, we use a simplified set of transitions in the hydrogen molecule proposed in [22], giving a van der Waals interaction coefficient of $C_6 = 3.08 \times 10^4$ au (atomic units) for both the 2S-6P$_{1/2}$ and 2S-6P$_{3/2}$ transitions. The close-to-resonance term is found to be negligible due to the small oscillator strength of the corresponding transitions in the hydrogen molecule. In addition, the close-to-resonance energy levels in the molecule may even suppress the pressure shift via transfer of excitation from the hydrogen atom to the molecule during the collision. The resulting pressure shift from intra-beam collisions with hydrogen molecules depends on the atoms' speed but is at most -1 Hz for the speeds relevant here (<1000 m/s). The resulting pressure shift from the background gas of room-temperature hydrogen molecules, on the other hand, is mostly independent of speed below 1000 m/s, and is at most -11 Hz (assuming that the background gas exclusively consists of hydrogen molecules). For both cases, the contribution from the van der Waals interaction dominates over that from the quadrupole electric field of the molecules.

Similarly (and similar to [23]), we estimate the pressure shift from water in the background gas, since the dipole moment of water molecules leads to a comparatively long-range and strong interaction with hydrogen atoms. We find a pressure shift of at most -3 Hz for the estimated partial pressure of water (7×10^{-8} mbar) inside the spectroscopy region.

Based on these results, we account for the pressure shift of the 2S-6P$_{1/2}$ and 2S-6P$_{3/2}$ transitions by including a 0.02 kHz uncertainty (assumed to be fully correlated between the two transitions) in $\nu_{1/2}$ and $\nu_{3/2}$.

2.5 Sampling bias

A sampling bias, i.e., a dependence of the extracted resonance frequency on the choice of line sampling, can arise when the line shape model used to fit the experimental line shape (and thereby extract the resonance frequency) does not match the experimental line shape exactly. This is the case here, as the Voigt and Voigt doublet line shape models we use for fitting do not include non-Gaussian (or non-Lorentzian) broadening and saturation effects, which are symmetric about the line center, nor do they include asymmetries from the LFS and QI, all of which are present in the experiment. Both symmetric and asymmetric deviations between the line shape fits and the experimental data are clearly visible in the (averaged) fit residuals (see Extended Data Fig. 1), with symmetric deviations dominating over asymmetric ones. Our simulations, on the other hand, include all the above effects. The better agreement of the simulations with the experimental line shapes is visible in Extended Data Fig. 1, which shows similar residuals for line shape fits to the simulations and for line shape fits to the experimental data.

To estimate the sampling bias, we therefore use our simulated line shapes, which are sampled and fit in the same way as the experimental line shapes. We distinguish two cases: a sampling bias for a line shape with asymmetric deviations sampled symmetrically about its unperturbed line center (i.e., the line center in the absence of asymmetric deviations), and a sampling bias for a line shape with generally both asymmetric and symmetric deviations sampled not symmetrically but offset from its unperturbed line center. Since we define the corrections for the LFS and the

QI shift as the resonance frequency from a fit to the corresponding simulations, the first case is intrinsically included in those corrections. For the second case, we apply a frequency offset to the sampling of the simulations. The resulting bias is linear in, but of opposite sign to, the frequency offset (for offsets $\lesssim 20\,\text{kHz}$), and identical within 10 Hz for the LFS simulations, QI simulations, and simulations using a simple model that includes non-Gaussian broadening and saturation, but not LFS or QI asymmetries. Hence, the bias is dominated by symmetric deviations, and we use the simple model to estimate it. We find the average frequency offset for each data group from the difference between the center laser frequency and the transition frequencies determined in this work. The average frequency offsets are mostly positive (caused by drift of the spectroscopy laser frequency) and reach at most 15 kHz, while the resulting bias reaches at most $-0.19\,\text{kHz}$. Overall, we estimate the sampling bias to be $-0.04\,\text{kHz}$ and $-0.08\,\text{kHz}$ for the 2S-6P$_{1/2}$ and 2S-6P$_{3/2}$ transitions, respectively. We include these estimates as uncertainties for $\nu_{1/2}$ and $\nu_{3/2}$ and treat them as fully correlated between the two transitions.

2.6 Signal background

We observe a signal background y_0, i.e., a signal when the 2S-6P spectroscopy laser is off resonance, on both detectors. The relative signal background y_0/A, where A is the line amplitude above the background when the spectroscopy laser is on resonance, ranges from 3 % to 12 % for the different data groups, and scales approximately inversely with spectroscopy laser power. It is approximately constant for the different velocity groups, the same for both detectors, and consistent over freezing cycles (and therefore atomic beam offset angle alignments). We attribute the signal background to Lyman-α photons emitted by the decay of metastable 2S atoms, as it is only present when atomic hydrogen flows into the nozzle and the 1S-2S preparation laser is on resonance (the dark count rate of the detectors when no 2S atoms are present is negligible).

The spontaneous decay of 2S atoms to the 1S ground level (by two-photon emission with a rate of $8.2\,\text{s}^{-1}$) inside the 52-mm-diameter 2S-6P spectroscopy region does not contribute substantially to the observed signal background. This is because, while the ratio of decayed 2S atoms to 2S atoms that have been excited to the 6P level can reach up to 50 % of the observed value of y_0/A (see Tables 5.1 and 5.4 of [1]), the detection efficiency of the emitted 243-nm photons is approximately four orders-of-magnitude lower than for the signal (Lyman-ϵ) photons.

External electric fields can lead to one-photon (Lyman-α) decay, or quenching, of 2S atoms through mixing of the 2S and 2P levels (with Lyman-α photons having a five-fold lower detection efficiency than Lyman-ϵ photons). The observed stray electric fields (strength below 1 V/m) inside the spectroscopy region, however, only lead to a one-photon decay rate of $3 \times 10^{-3}\,\text{s}^{-1}$, much too low to explain the signal background. Larger stray fields might be present at the apertures of the Faraday cage where the atomic beam enters and exits the spectroscopy region, but we have not characterized the stray fields outside the spectroscopy region.

Collisions with nearby particles can likewise quench the 2S atoms or deflect them (by angles $>0.1\,\text{rad}$) towards surfaces inside the spectroscopy region, where they quench upon impact. The collision partners and relevant numbers are discussed for the closely-related pressure shift in Section 2.4. We estimate that intra-beam collisions with 1S atoms and molecular hydrogen can only explain 0.4 % and 2 %, respectively, of the signal background. We find the dominant process to be collisional quenching by the quadrupole electric field of the hydrogen molecules [24] in the room-temperature background gas. However, the background gas density inside the spectroscopy region only explains $\approx$10 % of the observed signal background. We hypothesize that the bulk of the signal background stems from 2S atoms rapidly being quenched by molecular hydrogen as they leave the spectroscopy region and enter the outer vacuum region through a 30-mm-long differential pumping tube (see Section 4.2 of [1]), as the background gas pressure in the outer vacuum region is 30-fold higher than in the spectroscopy region but likewise dominated by molecular hydrogen.

This hypothesis is supported by the finding that the relative signal background scales linearly with the background gas pressure. As the fraction of molecular hydrogen freezing inside the nozzle depends strongly on temperature, we observed this scaling by varying the nozzle temperature within 3.7 K. . . 6 K. This changed the background gas pressure and the relative signal background

by more than an order of magnitude (see Section 4.5.2 of [1]). Furthermore, as the background pressure of water is not expected to strongly vary with the nozzle temperature, this test excludes quenching by water molecules as a dominant source for the signal background. For the data set presented here, for which the nozzle was held at constant temperature and the background gas pressure varied by less than 15 % (relative standard deviation), we find a significant correlation of the relative signal background with the pressure ($r = 0.50$ and $r = 0.29$ for the spectroscopy region and outer vacuum region, respectively). There is no significant correlation with the Doppler-free transition frequency ν_{e}.

The hypothesis is also consistent with the relative signal background being constant over velocity groups, as the speed of the background gas particles is always much larger than that of the 2S atoms, leading to an approximately constant relative speed. In this case, the collision probability scales with the time spent in the background gas, and therefore with $1/v$, where v is the atom's speed. Since the 6P excitation probability also approximately scales as $1/v$ for our experimental conditions, this results in a relative signal background independent of v, i.e., the relative signal background is identical for the different velocity groups.

A signal background that is not constant with spectroscopy laser detuning could potentially lead to line shifts. To constrain such shifts, we estimate the linear slope of the signal background from the difference in the signal at the maximum detunings ($\Delta = \pm 50\,\mathrm{MHz}$), where we take into account any signal differences expected from the LFS or QI shift. We then artificially remove this linear slope from the signal of each line scan and re-analyze the data. Overall, we find statistically insignificant shifts of, respectively, $-40\,\mathrm{Hz}$ and $-30\,\mathrm{Hz}$ for the 2S-6P$_{1/2}$ and 2S-6P$_{3/2}$ transitions. We include these estimates as uncorrelated uncertainties for $\nu_{1/2}$ and $\nu_{3/2}$.

2.7 Laser systems and frequency standard

The two laser systems used in the measurement, the 2S-6P spectroscopy laser at 410 nm and the 1S-2S preparation laser at 243 nm, share a similar design. Both systems use external cavity diode lasers (cavity length ∼20 cm, described in [25]) as seed lasers in the infrared at 820 nm and 972 nm. The lasers are phase-stabilized to high-finesse Fabry-Pérot cavities, which reduces the linewidth to a few Hz [26]. This narrow carrier sits on a weak but broad (∼1 MHz) noise pedestal [25, 26]. The linewidth and the spectral purity of the lasers are monitored with a beat note between them using a low-noise, Er-doped fiber optical frequency comb as transfer oscillator [27, 28]. After power amplification with a tapered amplifier, the light is frequency doubled (frequency doubled twice) to 410 nm (243 nm) for the spectroscopy (preparation) laser.

Asymmetries in the noise pedestal of the spectroscopy laser may lead to line shifts. We obtain an upper limit of 0.07 kHz by artificially introducing an asymmetry of 10 % into the measured noise pedestal [25], numerically convolving it with the 2S-6P line shape, and fitting the result. We include this upper limit as a fully correlated uncertainty for the 2S-6P$_{1/2}$ and 2S-6P$_{3/2}$ transitions.

The frequency comb is used to compare the optical frequencies of the 2S-6P spectroscopy laser and the 1S-2S preparation laser to the microwave frequency of a passive hydrogen maser (see Section 4.8 of [1] and Section 3.2.3 of [29]). To this end, the repetition rate and carrier–envelope offset frequency of the frequency comb, and the beat notes between the frequency comb modes and the lasers, are continuously measured using frequency counters referenced to the maser. The frequency of the hydrogen maser, in turn, is continuously compared against the caesium frequency standard, which is the basis of the unit of hertz in the International System of Units (SI), using a global navigation satellite system (GNSS) receiver (with the gravitational redshift taken into account). This comparison leads to corrections of the transition frequencies by 0.02 kHz (2.5×10^{-14}) and 0.01 kHz (1.9×10^{-14}) for the 2S-6P$_{1/2}$ and 2S-6P$_{3/2}$ transitions, respectively. The total fractional frequency uncertainty of the comparison (between the frequency comb, hydrogen maser, and caesium standard) is estimated as 1×10^{-14}, corresponding to 7 Hz for both transitions (here rounded to 0.01 kHz and assumed to be fully correlated).

The fundamental frequencies (in the infrared) of the spectroscopy and preparation lasers drift, on average, by 5.4(2.9) kHz/day and 2.6(2.7) kHz/day, respectively (standard deviation over measurement days in parentheses). During the approximately 1 min required to record a single line

scan, this corresponds to an insignificant drift at the atomic transition frequencies of 8 Hz and 15 Hz. We determine the laser frequencies by linear fits to 1-hour-long windows of the frequency counter data, resulting in a statistical uncertainty below 10 Hz for each window, and therefore negligible compared to the statistical uncertainty of the line shape fits.

2.8 Recoil shift

Energy and momentum conservation require the energy of a photon driving an atomic transition of frequency ν to be larger than the corresponding transition energy $h\nu$ by the recoil shift. The recoil shift for the for 2S-6P$_{1/2}$ and 2S-6P$_{3/2}$ transitions can be written as

$$\Delta\nu_{\mathrm{rec}} = \frac{h}{2m_{\mathrm{H}}}\left(\frac{\nu}{c}\right)^2 \approx 1176.03\,\mathrm{kHz}, \tag{3}$$

The transition frequency ν is identical at the required level of accuracy for both transitions and is known with much lower uncertainty than needed here. Likewise, the mass of the hydrogen atom can be found by $m_{\mathrm{H}} \approx m_{\mathrm{e}}(m_{\mathrm{p}}/m_{\mathrm{e}} + 1 + \alpha^2/2)$, where m_{e} is the electron mass, with sufficient accuracy. Corrections to $\Delta\nu_{\mathrm{rec}}$ from wavefront curvature and the Gouy phase [30] amount to at most 0.1 Hz and are neglected here. $\nu_{1/2}$ and $\nu_{3/2}$ have been corrected for the recoil shift by subtracting $\Delta\nu_{\mathrm{rec}}$.

References

[1] Maisenbacher, L. *Precision Spectroscopy of the 2S-nP Transitions in Atomic Hydrogen*. Ph.D. thesis, Ludwig-Maximilians-Universität München (2020). Revised version, arXiv:2512.20543, doi:10.48550/arXiv.2512.20543

[2] Haas, M. *et al.* Two-photon excitation dynamics in bound two-body Coulomb systems including ac Stark shift and ionization. *Physical Review A* **73**, 052501 (2006). doi:10.1103/PhysRevA.73.052501

[3] Wolfram Research, Inc. Mathematica, Version 14.2.

[4] Hairer, E., Wanner, G. & Nørsett, S. P. *Solving Ordinary Differential Equations. Nonstiff Problems* 2 edn, Vol. 8 of *Springer Series in Computational Mathematics* (Springer, Berlin, Heidelberg, 1993). doi:10.1007/978-3-540-78862-1

[5] Hairer, E. & Wanner, G. *Solving Ordinary Differential Equations. Stiff and Differential-Algebraic Problems* 2 edn, Vol. 14 of *Springer Series in Computational Mathematics* (Springer, Berlin, Heidelberg, 1996). doi:10.1007/978-3-642-05221-7

[6] Horbatsch, M. & Hessels, E. A. Tabulation of the bound-state energies of atomic hydrogen. *Physical Review A* **93**, 022513 (2016). doi:10.1103/PhysRevA.93.022513

[7] Bethe, H. A. & Salpeter, E. E. *Quantum Mechanics of One- and Two-Electron Atoms* (Springer, 1957). doi:10.1007/978-3-662-12869-5

[8] Wiese, W. L. & Fuhr, J. R. Accurate atomic transition probabilities for hydrogen, helium, and lithium. *Journal of Physical and Chemical Reference Data* **38**, 565–720 (2009). doi:10.1063/1.3077727

[9] Horbatsch, M. & Hessels, E. A. Shifts from a distant neighboring resonance. *Physical Review A* **82**, 052519 (2010). doi:10.1103/PhysRevA.82.052519

[10] Udem, Th. *et al.* Quantum interference line shifts of broad dipole-allowed transitions. *Annalen der Physik* **531**, 1900044 (2019). doi:10.1002/andp.201900044

[11] Beyer, A. *et al.* The Rydberg constant and proton size from atomic hydrogen. *Science* **358**, 79–85 (2017). doi:10.1126/science.aah6677

[12] Mølmer, K., Castin, Y. & Dalibard, J. Monte Carlo wave-function method in quantum optics. *Journal of the Optical Society of America B* **10**, 524–538 (1993). doi:10.1364/JOSAB.10.000524

[13] Fleischhauer, M. & Marangos, J. P. Electromagnetically induced transparency: Optics in coherent media. *Reviews of Modern Physics* **77**, 633–673 (2005). doi:10.1103/RevModPhys.77.633

[14] Farley, J. W. & Wing, W. H. Accurate calculation of dynamic Stark shifts and depopulation rates of Rydberg energy levels induced by blackbody radiation. Hydrogen, helium, and alkali-metal atoms. *Physical Review A* **23**, 2397–2424 (1981). doi:10.1103/PhysRevA.23.2397

[15] Wirthl, V. *et al.* Improved active fiber-based retroreflector with intensity stabilization and a polarization monitor for the near UV. *Optics Express* **29**, 7024–7048 (2021). doi:10.1364/OE.417455

[16] Wirthl, V. *et al.* Improved active fiber-based retroreflector with intensity stabilization and a polarization monitor for the near UV: Erratum. *Optics Express* **30**, 7340–7341 (2022). doi:10.1364/OE.454374

[17] Matveev, A., Kolachevsky, N., Adhikari, C. M. & Jentschura, U. D. Pressure shifts in high-precision hydrogen spectroscopy: II. Impact approximation and Monte-Carlo simulations. *Journal of Physics B: Atomic, Molecular and Optical Physics* **52**, 075006 (2019). doi:10.1088/1361-6455/ab08e1-1

[18] Jentschura, U. D. & Adhikari, C. M. Long-range interactions for hydrogen: 6P-1S and 6P-2S systems. *Atoms* **5**, 48 (2017). doi:10.3390/atoms5040048

[19] Jentschura, U. D., Adhikari, C. M., Dawes, R., Matveev, A. & Kolachevsky, N. Pressure shifts in high-precision hydrogen spectroscopy. I. Long-range atom-atom and atom-molecule interactions. *Journal of Physics B: Atomic, Molecular and Optical Physics* **52**, 075005 (2019). doi:10.1088/1361-6455/ab08cc-1

[20] Sobel'man, I. I., Vainshtein, L. A. & Yukov, E. A. *Excitation of Atoms and Broadening of Spectral Lines* 2 edn. Springer Series on Atomic, Optical, and Plasma Physics (Springer, Berlin, Heidelberg, 1995). doi:10.1007/978-3-642-57825-0

[21] Poll, J. D. & Wolniewicz, L. The quadrupole moment of the H_2 molecule. *The Journal of Chemical Physics* **68**, 3053–3058 (1978). doi:10.1063/1.436171

[22] Victor, G. A. & Dalgarno, A. Dipole properties of molecular hydrogen. *The Journal of Chemical Physics* **50**, 2535–2539 (1969). doi:10.1063/1.1671412

[23] Brandt, A. D. *et al.* Measurement of the $2S_{1/2}$-$8D_{5/2}$ transition in hydrogen. *Physical Review Letters* **128**, 023001 (2022). doi:10.1103/PhysRevLett.128.023001

[24] Slocomb, C. A., Miller, W. H. & Schaefer, H. F. Collisional quenching of metastable hydrogen atoms. *The Journal of Chemical Physics* **55**, 926–932 (1971). doi:10.1063/1.1676163

[25] Kolachevsky, N. *et al.* Low phase noise diode laser oscillator for 1S-2S spectroscopy in atomic hydrogen. *Optics Letters* **36**, 4299–4301 (2011). doi:10.1364/OL.36.004299

[26] Alnis, J., Matveev, A., Kolachevsky, N., Udem, Th. & Hänsch, T. W. Subhertz linewidth diode lasers by stabilization to vibrationally and thermally compensated ultralow-expansion glass Fabry-Pérot cavities. *Physical Review A* **77**, 053809 (2008). doi:10.1103/PhysRevA.77.053809

[27] Holzwarth, R. *et al.* Optical frequency synthesizer for precision spectroscopy. *Physical Review Letters* **85**, 2264–2267 (2000). doi:10.1103/PhysRevLett.85.2264

[28] Giunta, M. *et al.* Real-time phase tracking for wide-band optical frequency measurements at the 20th decimal place. *Nature Photonics* **14**, 44–49 (2020). doi:10.1038/s41566-019-0520-5

[29] Wirthl, V. *Precision Spectroscopy of the 2S-6P Transition in Atomic Deuterium.* Ph.D. thesis, Ludwig-Maximilians-Universität München (2022). doi:10.5282/edoc.31589

[30] Bade, S., Lionel Djadaojee, Manuel Andia, Pierre Cladé & Saïda Guellati-Khelifa. Observation of extra photon recoil in a distorted optical field. *Physical Review Letters* **121** (2018). doi:10.1103/PhysRevLett.121.073603
